# Effect of silver diamine fluoride activation on bond strength to root dentin

**DOI:** 10.1186/s12903-023-03457-2

**Published:** 2023-10-09

**Authors:** Sara Elmallah, Ahmed Abdou, Amr Rizk, Citra Kusumasari, Reem Ashraf

**Affiliations:** 1 Department of Endodontics, Faculty of Dentistry, Fayoum University, Fayoum, Egypt; 2 Department of Prosthetic Dentistry, Biomaterials Division, Faculty of Dentistry, King Salman International University, El Tur, South Sinai Egypt; 3 Faculty of Dentistry, Al-Ayen University, Thi-Qar, Iraq; 4 Department of Prosthetic Dentistry, Fixed Prosthodontics Division, Faculty of Dentistry, King Salman International University, El Tur, South Sinai Egypt; 5 Department of Conservative Dentistry, Faculty of Dentistry, Universitas Indonesia, Jakarta, Indonesia

**Keywords:** Silver diamine fluoride, Endodontic treatment, Irrigation, Push-out bond strength, Fiber post

## Abstract

**Background:**

To investigate the effect of silver diamine fluoride (SDF) application and activation on the bond strength of gutta-percha to dentin and resin bonded post to dentin.

**Methods:**

Thirty-six human premolar teeth were used. The coronal part tooth was removed, and endodontic mechanical preparation was performed for all the teeth. The teeth were divided according to final rinse protocol (*n* = 9) as follows: Control group; no SDF application, SDF/NA; 38% SDF as a final rinse with no activation, SDF/MDA; 38% SDF as a final rinse with manual dynamic activation and SDF/US; 38% SDF as a final rinse with ultrasonic activation. Root canal obturation using lateral condensation technique followed by fiber post insertion after 48 h in the root canal after corresponding preparations. The roots were cut with a low-speed precision saw creating 2 mm thickness sections. A total of 4 sections were obtained from each tooth, 2 coronal specimens (with post) and 2 apical specimens (with Gutta percha). Each specimen was subjected to push-out bond strength test with a universal testing machine. Data were analyzed using two-way ANOVA.

**Results:**

The Push-out bond strength at the apical root section was significantly higher in SDF/MDA and SDF/US groups compared to control group. While for the coronal part, all SDF treated specimen showed reduced Push-out bond strength.

**Conclusion:**

SDF application as final rinse may reduce the bonding performance between fiber post and dentine. Activation with manual and ultrasonic methods improved the bond strength at the apical root section.

## Background

The success of any endodontic treatment relies on achieving a clean, bacteria free and tightly sealed root canal obturation that will prevent possible complications such as apical periodontitis and/or failure of the endodontic treatment [1]. Root canal disinfection can be achieved by antimicrobial solutions and intracanal medicaments [2, 3].

Conventionally, irrigants are delivered by a syringe and a needle, but this technique is unable to create an adequate flow inside narrow areas such as lateral canals and isthmuses [4, 5]. As a result, a considerable amount of intact biofilm may persist following the chemo-mechanical preparation, which is associated with the root canal treatment failure [6, 7].

The adjunctive use of medicaments as a final rinse by either conventional method or by the activation methods have been proposed to improve the cleaning and disinfection of canal ramifications. Activation methods includes manual agitation and ultrasonic activation [8]. The latter being the most popular and able to remove the smear layer as it is based on the principle of cavitation and acoustic streaming that makes it more effective in than conventional needle irrigation [9–13].

Sodium hypochlorite (NaOCl) had been considered as the gold standard in endodontics canal irrigation [14]. However, conflicting reports on how the bond strength to dentin is affected by the use of NaOCl with the variation in its form, concentration and time of application [15, 16].

Recently, silver diamine fluoride (SDF) introduction into the preventive and restorative dentistry highlighted its potential use as a potent antimicrobial agent [17]. The use of SDF as an irrigation protocol was investigated in literature to ensure that a clean sterile root canal is ready for receiving the targeted filling materials and would provide a better space to receive the appropriate restoration for the canal [18].

Endodontically treated teeth usually have a limited tooth structure therefore the placement of a post is somehow mandatory for a better clinical outcome. Fiber posts particularly require removal of smear layer and adequate bonding to dentin to achieve a strong seal that is critical for longevity [19].

The use of SDF as a final rinse may alter the cementation protocol with fiber post. Additionally, the activation methods can affect the SDF penetration which can affect the bond strength of both gutta perch and post, thus the null hypothesis of the current study was that application of SDF without or with activation will not affect the bond strength of gutta-percha to dentin nor resin bonded post to dentin.

## Materials and methods

### Teeth selection

A total of 36 human premolar teeth with a single root canal were collected from the outpatient clinic of the oral and maxillofacial surgery department (Fayoum University) after taking the patient approval for using his extracted premolar/s for the research use. The extraction was due to orthodontic reasons. Ethical approval was obtained from Research Ethics Committee, Faculty of Dentistry, Ahram Canadian University. Research Number: IRB00012891#52. All collected teeth had an average root length of 15 mm (and up to 16 mm) that was measured from the root apex to the cemento-enamel junction of the buccal surface. The root length was verified using a digital caliper (Mituoyo CD-15C; Mitutoyo, Kanagawa, Japan). The teeth were selected with the inclusion criteria as follows: a) identifiable single canals, b) caries free, c) no internal/external resorption, d) no calcifications, e) no fractures/crack lines, f) straight roots of similar length and g) fully formed apices. The conformance to these criteria was verified by visual and radiographic inspection that was taken by the paralleling technique in both buccolingual and mesiodistal directions [20]. Any tooth that failed to fulfill the forementioned criteria was replaced.

### Teeth grouping

The collected teeth were allocated into 4 main groups (*n* = 9 each) according to the irrigation protocol and were stored in saline with 1% thymol at room temperature in separate vials until further usage. All groups were irrigated with 2.5% NaOCl using a conventional syringe irrigation (plastic syringe 3 ml,23 gauge) followed by normal saline irrigation.

The groups were divided according to final rinse protocol (*n* = 9) as follows: Control group; no SDF application, SDF/NA; 38% SDF as a final rinse with no activation (NA), SDF/MDA; 38% SDF as a final rinse with manual dynamic activation (MDA) and SDF/US; 38% SDF as a final rinse with ultrasonic activation (US).

### Root canal preparation and obturation

The coronal part of each tooth was removed 1 mm above the cementoenamel junction (CEJ) from the highest peak of CEJ curvature of the mesial tooth surface using a diamond disc (D&Z, Germany). All endodontic procedures were performed by the same experienced operator.

A #10 K-file (Dentsply Millefere, OK, USA) was placed within the canal to the extent that the file tip was visible from the end of the apex, to ensure the canal patency and to establish the working length 1 mm shorter than this length.

The canals were prepared using the crown-down technique where the rotary HyFlex EDM files (Coltene/Whaledent, Allstätten, Switzerland) were used sequentially as follows: A 25/0.12 file to shape the cervical part, the 10/0.05 file (Glidepath files) was used for the initial exploration of the apical part and a 25/ ~ file variable taper was used then file 40/0.04 was used for finishing the preparation.

The files were used on an X-Smart engine (Dentsply-Maillefer, OK, USA) operating at 500 rpm with a torque of up to 2.5 Ncm, except for the Glidepath files, which were used at 300 rpm with a torque of up to 1.8 Ncm. EDTA gel was used as a chelating agent during the instrumentation. In between each file change, the canals were irrigated with 3 ml of 2.5% NaOCl by using a 23-gauge needle which was placed 2 mm before the working length. Each instrument was replaced according to the manufacturer’s recommendations. Then irrigation with saline was done (to remove all the remnants of NaOCl) followed by using the SDF (38% SDF, ToothMate, Mansoura, Egypt) as a final rinse using a 23-gauge needle for SDF/NA group. The irrigation of the control group was the same as the forementioned protocol without the SDF application as a final rinse.

The activation of the SDF was done differently according to the groups. In SDF/NA group; no activation was done while in SDF/MDA group; the Manual Dynamic Activation technique (MDA) was done by using the master cone gutta-percha 1 mm set back from the working length, 2 mm in-and-out movement inside the irrigating solution for 15 s and in SDF/US group; ultrasonic activation for the SDF was performed by using a size 20/0.00 taper and 21 mm Irrisafe file (Acteon Satelec, Merignac, France) attached to an Ultrasonic device (P5 Newtron XS, Acteon Satelec). The power setting was 9/20 (45%) following the manufacturer instructions. The file was placed 2 mm from the apical end point of the root canal, and the activation time was done for 15 s.

Lateral condensation technique along with epoxy resin-based root canal sealer (Adseal, Meta Biomed, Korea) was used for obturating the canals. Tug-back in the master cone was confirmed by tactile sense to create and maintain apical seal.

### Post space preparation and cementation

Teeth were stored in 100% humidity at 37 °C for 48 h prior to post space preparation to ensure complete setting of sealer. Subsequently, the calibrating drill was used to prepare space for FiberKleer 4X fiber post (Pentron, USA) till size 3 up to the length of 10 mm from the surface of the cut crown by the aid of drill stopper leaving at least 5 mm of gutta-percha intact inside the canal. A new drill set was used to prepare post space after every five specimens. The post space was then irrigated with saline and dried with paper point. The post space length and remining gutta percha length was confirmed using paralleling technique radiograph taken in buccolingual and mesiodistal directions [20].

A size 3 (diameter 1.5 mm at tip) fiber post was placed inside the canal to check its seating and length achievement by marking it at 10 mm. After making sure that the post is completely seated at 11 mm length (including 1 mm above the cement-enamel junction) it was removed and cut to the marked length with a precision cutting saw (IsoMet 4000, Buehler, USA). Posts were then cleaned with 70% alcohol for 30 s, rinsed with distilled water, air-dried, and stored in a sterilized pouch until fiber post cementation was performed.

All fiber posts were cemented using dual cured self-adhesive resin cement (Breeze, Pentron, USA). The cement was introduced into the canal by forceful injection using intra-canal tips until it oozed outside of the canal and then the fiber post was inserted into the canal until complete seating up to the prepared length. Excess cement was removed using a micro brush and the cement was light cured for 40 s from coronal position. After the cementation procedures, all specimens were stored in sterile saline in a light-proof box for one week at 37 °C to prevent dehydration.

### Push-out bond strength evaluation for both fiber post and gutta-percha

**Fig. 1:**
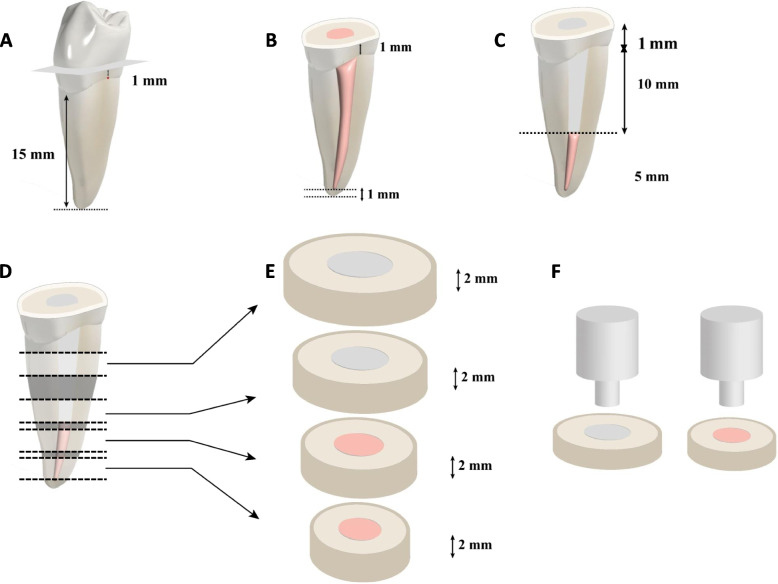
Diagrammatic illustration of the tooth selection and preparation. **A** Premolar tooth with root length of 15 mm from the apices to the cervical line. **B** removal of the coronal dentin 1 mm above the CEJ. **C **Perpartion of post cavity of 10 mm. **D **Root sectioning position. **E** four specimens were cut from post-space (2 specimens) and gutta percha (2 specimen). **F** Pin used for push-out bond strength testing and position on the center of the specimen

Each root was sectioned perpendicular to the long axis with a low-speed precision cutting saw (IsoMet 4000, Buehler, USA) under constant water cooling to create 4 specimens of 2 mm thickness in both fiber post (2 specimens) and gutta-percha (2 specimens). Creating a total of 18 post specimens and 18 gutta-percha specimens in each tested group. To check the specimen dimensions before push-out bond strength testing a digital caliper (Mituoyo CD-15C; Mitutoyo) was used and the exact dimension was used for bond strength calculations. The root dimensions and specimen preparation steps are listed in Fig. 1.

The push-out test was performed by using the universal testing machine (Model 3345; Intsron Industrial Products, Norwood, MA, USA) at a crosshead speed of 1 mm/minute, using a pin (diameter, 1.0 mm for fiber post and 0.6 mm for gutta-percha) on the center of the apical aspect of the post surface and gutta-percha in an apical-coronal direction respectively, without stressing the surrounding dentinal walls. The peak force (N) required to extrude the post and gutta-percha from the root slice was recorded. To express the bond strength in MPa, the load at failure (N) was divided by the area of the bonded interface, which was calculated with the following formula:

$$A=\pi \left({\mathrm{r}}_{1}+{\mathrm{r}}_{2}\right)\sqrt{{\left({r}_{1}-{r}_{2}\right)}^{2}+{h}^{2}}$$

where π was the constant 3.14, r_1_ was the coronal post or gutta-percha radius, r_2_ was the apical post or gutta-percha radius, and h was the thickness of the slice in millimeters.

### Fiber post mode of failure analysis

Fiber post failure modes were assessed using a stereo microscope at 10 × magnification. The failure modes were classified as: Mode A; adhesive (total dislocation of post and resin cement), Mode B; cohesive (failure associated to a single body, i.e., dentin, post, or cement) and Mode C; mixed (post dislocation with resin cement remnants).

### Statistical analysis

Sample size was calculated based on data extracted from previously published paper [21]. For comparison between control and agitation group the true difference is 1.2 MPa for push-out bond strength and the effect size is f = 1.448. The minimum sample size is 9 in each group will be sufficient to detect 80% power. Sample size was calculated using G*Power 3.1.9.7. Data showed normal distribution when checked using Kolmogorov- Smirnov test. Two-way ANOVA used to compare between different tested groups and root section. Multiple comparisons were done with Tukey’s HSD test. A significant level was set at *p* = 0.05 (SPSS IBM, version 26, Armonk, NY, USA).

## Results

Results of two-way ANOVA and push out bond strength presented in Tables 1 and 2. Two-way ANOVA showed that different groups and root section had significant effect on the mean push-out bond strength (*p* < 0.001 and 0.012, respectively). The interaction between both variables resulted also in a significant effect on the mean push-out bond strength at *p* < 0.001. For Gutta-percha (apical root section), Control group showed the lowest significant push-out bond strength compared to SDF/MDA (*p* = 0.022) and SDF/US (*p* = 0.011).

**Table 1:** Two-Way ANOVA for push-out bond strength

Source	Type III Sum of Squares	df	Mean Square	F	Sig
Root section	123.752	1	123.752	30.336	< 0.001
Groups	50.445	3	16.815	4.122	0.012
Root section ✕ Groups	204.221	3	68.074	16.687	< 0.001

**Table 2:** Data for push-out bond strength for different tested groups

	Gutta percha	Post	Mean Difference [95% CI]	*p*-value
SDF/NA	3.97^ab^ ± 1.57	8.27^b^ ± 3.85	-4.29[-7.72 to -0.87]	< 0.001
SDF/MDA	4.69^a^ ± 1.48	3.56^c^ ± 0.83	1.14[-0.26 to 2.54]	0.298
SDF/US	4.98^a^ ± 1.73	4.61^c^ ± 2.15	0.37[-1.89 to 2.64]	0.731
Control	1.72^b^ ± 0.61	11.9^a^ ± 1.09	-6.12[-8.98 to -3.26]	< 0.001
*p*-value	0.012	< 0.001		

*CI* Confidence interval. Different letters within each column indicate significant difference at *p* < 0.05 (Adjusted Tukey’s HSD)

Insignificant differences resulted between SDF/NA and all other groups. For Post (coronal root section), control group showed the highest significant bond strength compared to SDF/NA (*p* = 0.043), SDF/MDA (*p* < 0.001), and SDF/US (*p* = 0.001). SDF/MDA and SDF/US showed an insignificant difference between each other’s (*p* = 0.849). SDF/NA showed the highest significant bond strength compared to SDF/MDA (*p* = 0.008), and SDF/US (*p* = 0.048).

Significant difference resulted between root sections (Gutta-percha and post) for control (*p* < 0.001) and SDF/NA (*p* < 0.001), in both groups the coronal portion showed higher push-out bond strength. While insignificance difference resulted between coronal and apical root section for SDF/MDA (*p* = 0.298) and SDF/US (*p* = 0.731).

**Fig. 2:**
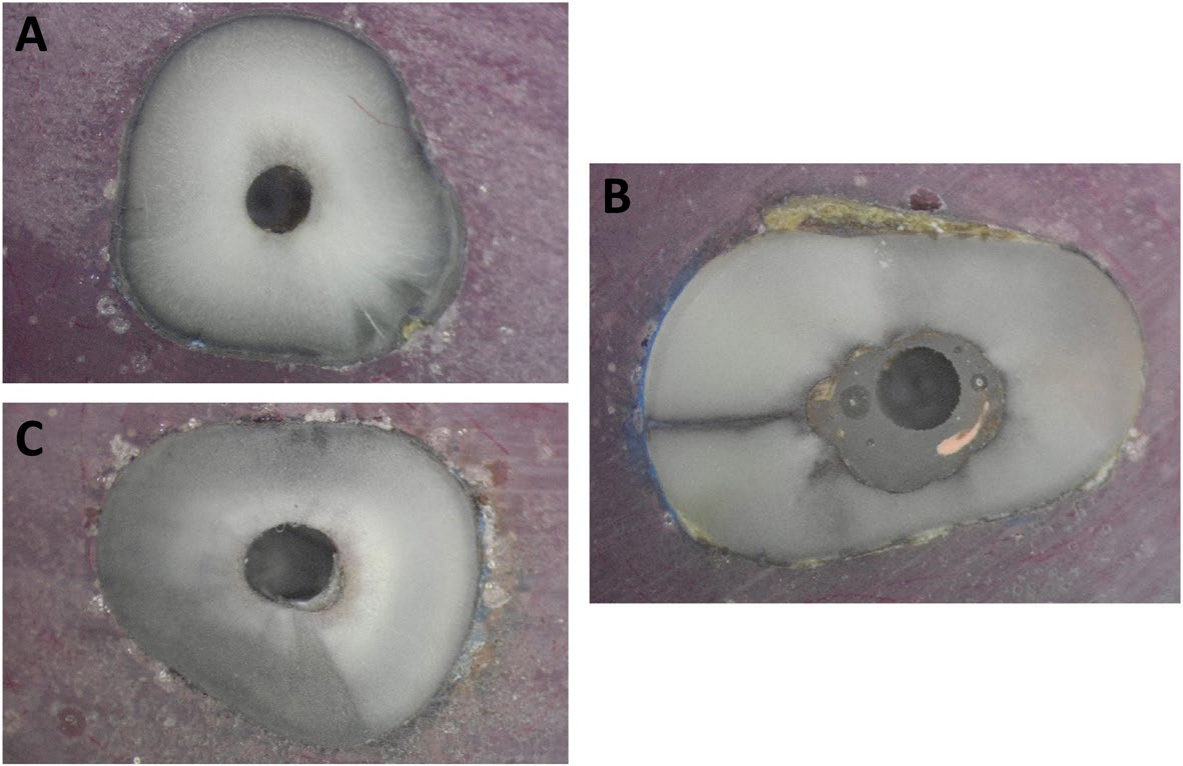
Representative images for the failure mode analysis in the root post section. **A** adhesive failure (total dislocation of post and resin cement), **B** cohesive failure (associated to a single body, i.e., dentin, post, or cement) and **C** mixed failure (post dislocation with resin cement remnants)

**Fig. 3:**
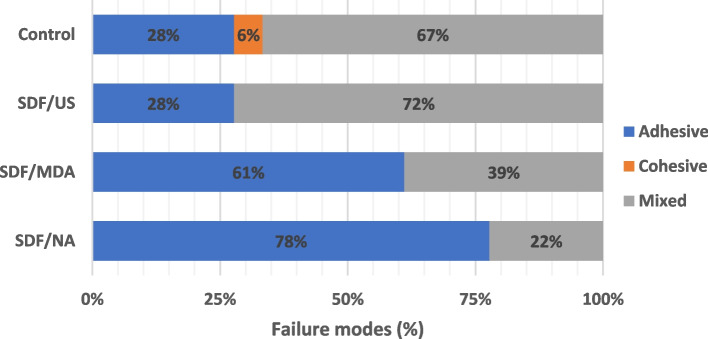
Stacked Bar chart showing distribution of failure modes

For failure mode analysis, a representative image for different scores for failure mode analysis is presented in Fig. 2. SDF/NA and SDF/MDA showed the highest adhesive failure (77.8 and 61.1%) and the lowest mixed failure (22.2 and 38.9%) compared to control and SDF/US which showed reverse behavior as shown in Fig. 3.

## Discussion

The current study investigated the effect of SDF as a final rinse in endodontically treated teeth with manual and ultrasonic activation. Based on the results of the current study, the null hypothesis was rejected as the bonding between gutta percha and dentin was improved at the apical one third for SDF activated groups. Moreover, the bonding performance between fiber post and dentine was decreased for SDF treated groups.

A variety of irrigation protocols were introduced for cleaning and shaping of root canals, claiming their potential to create a sterile chamber for gutta-percha and post insertion. The cleansing ability of such irrigant should not alter the surface or negatively affect the adhesive interface, Thus, after ensuring proper cleaning and thorough penetration into the canal, we must guarantee that the procedures did not compromise adhesion to gutta-percha or inadequate bonding to the post [22–24].

After irrigation of the canal the use of a final rinse with agents that may stimulate different characteristics as antimicrobial effect was found to be beneficial for root dentine disinfection, but its use may result in structural modifications of dentine, which could interfere with the bond strength to dentine.

The use of final rinse was investigated in previous studies to evaluate the harmful effects of the use of chemicals during endodontic treatment on the retention of posts to dentine claiming their potential to change the ratio of organic to inorganic components which in turn changed the biomechanical properties of dentine [25–27].

SDF has proven its antimicrobial activity and substantivity as well as its remineralization capacity for treatment of carious lesions [28]. Recently, it was used as a final rinse after root canal cleaning and shaping and had a pronounced effect on canal disinfection, but further investigations about its effect on bond strength had to be evaluated. [16] SDF was used in this study with different activation methods to improve its access to inaccessible areas as the apical region of the root with the presence of large number of accessory canals that is hard to penetrate without activation [29, 30]. Push-out test was the test of choice as it is a widely accepted method for recording the interfacial bond strength of endodontic materials to root dentin [29–31]. The bond strength of gutta-percha to the root dentine in the apical third of the canal was found to be higher for the control group (that was not rinsed with SDF) compared to SDF/MDA and SDF/US. These indicate the superior bond of the SDF activated groups to gutta-percha compared to control group.

The highest adhesive forces are mainly linked to the smear layer removal, that means when the organic debris is removed, and the collagen is exposed, and a strong seal is achieved [32–34].

The strong bond associated by SDF/MDA and SDF/US may be attributed to the ability of the root canal sealer to profoundly attach to the tubular dentine after being activated with manual agitation and ultrasonic activation that succeeded in removal of residual debris and smear layer which act as a physical barrier that prevents penetration of the epoxy resin sealer into the dentinal tubules [29].

Additionally, the epoxy sealer used in this study is capable of creating a covalent bond between epoxide (open circle) and the exposed amino groups in collagen, instead of degrading the collagen fibers that is incident by other types of irrigating solutions [35, 36].

Regarding the bond strength of root dentine to the post cemented by dual cured self-adhesive resin cement, for all the groups where SDF was used as a final rinse had a significantly lower bond strength in comparison to the control group, with the least values recorded by the activated groups SDF/MDA and SDF/US. The results with agreement with previous research which reported a decrease in the bond strength with root dentin after 38% SDF application with self-adhesive resin cement [37]. While another research reported an insignificant decrease in the bond strength, which may be attributed to the use of lower concentration (3.8%) SDF [38].

The deterioration of bond in this case might be related to the use of SDF in high concentration (38% that contains 253,900 ppm silver and 44,800 ppm fluoride) as SDF starts a process of remineralization within 24 h of interaction with dentine by deposition of fluoride and silver that reacts with the calcium phosphate of dentine hydroxyapatite forming a harder and more resistant fluorapatite crystals that are deposited in the dentinal tubules [39–41].

Moreover, in current study the teeth were stored for 48 h prior to post space preparation which might give a chance for the residual mineral crystals of the tooth to serve as nucleation sites for fluor-hydroxyapatite formation promoted by the alkaline characteristic of SDF that favors mineral deposition forming a more acid resistance smear layer [42, 43]. The later with the occluded dentinal tubules may prevent monomer penetration thus weakening of the interfacial bond. That was confirmed by the predominant adhesive failure mode in all groups treated with SDF.

The activation with both methods resulted in the least bond strength with resin cement and fiber post, which could be attributed to the better penetration of SDF into the dentinal tubules after activation which limits the penetration of the viscous resin cement [37] with the formation of silver crystals [17, 44–46] and hinder the polymerization of the self-adhesive resin [47, 48]. In addition the bonding failure of the activated groups was more pronounced than the non-activated group which confirms the idea of better penetration of SDF and crystal deposition that obliterated the mechanical interlocking of the adhesive resin. To our knowledge, the current study is the first to report the effect of the activation method of the SDF and its effect on the post cementation with self-adhesive resin cement which present an important finding about the activation methods and its’ effect on the bonding performance with radicular dentine.

Some limitations in the present study should be considered: (i) different concentrations of SDF might have different influence on dentine structure (ii) the use of different types of cement for bonding of post may have an altered effect. Additionally, further research is needed to investigate the penetration of SDF into dentine after usage as a final rinse with various activation methods.

## Conclusion

Within the limitation of this study, the use of SDF as a final rinse may adversely affect the bonding performance between fiber post and dentine. Meanwhile, it might enhance bond strength of gutta-percha to root dentine. SDF as a final rinse combined with manual activation and ultrasonic activation can improve the bonding in the apical root section of the endodontically treated tooth.

## Data Availability

The raw data required to reproduce these findings are available upon reasonable request from the corresponding author. The processed data required to reproduce these findings are available upon reasonable request from the corresponding author.
